# Spinal epidural lipomatosis – an easily ignored secondary intraspinal disorder in spinal kyphotic deformities

**DOI:** 10.1186/s12891-017-1467-7

**Published:** 2017-03-16

**Authors:** Zhen Zhang, Zhen Liu, Zezhang Zhu, Yong Qiu

**Affiliations:** Department of Spine Surgery, Nanjing Drum Tower Hospital of Nanjing University Medical School, Nanjing, China

**Keywords:** Spinal epidural lipomatosis, Spinal kyphotic deformities, Congenital kyphosis, Scheuermann kyphosis, Tuberculotic kyphosis

## Abstract

**Background:**

A previous study reported a high prevalence of spinal epidural lipomatosis (SEL) in patients with Scheuermann kyphosis (SK) and suggested that it may play a role in the pathogenesis of this disease. According to our observation, however, SEL occurs in other spinal kyphotic deformities as well. The aim of this study was to test the hypothesis that SEL commonly occurs in patients with different types of kyphotic deformities as a secondary intraspinal disorder.

**Methods:**

MR images of 16 patients with congenital kyphosis (CK), 40 patients with SK, 13 patients with tuberculotic kyphosis (TK), and 69 age- and sex-matched controls were retrospectively evaluated. The body mass index (BMI), kyphosis Cobb angle, and sagittal diameters of spinal epidural fat (EF) and the dural sac (DS) in the apical region (EF_A_, DS_A_) and non-kyphotic region (EF_N_, DS_N_) were measured. The EF ratios at the apical vertebral level (EFR_A_) and in the non-kyphotic region (EFR_N_) were calculated as EF / (EF + DS).

**Results:**

EF_A_ and EFR_A_ were significantly higher among patients with CK, SK, and TK than among controls (*P* < 0.05). Seven CK patients (43.8%), 8 SK patients (20.0%), and 11 TK patients (84.6%) fulfilled the diagnostic criteria for SEL, while only 6.3, 2.5, and 0% of patients in the control groups did (*P* = 0.019, 0.014, and < 0.001, respectively). Spearman’s correlation analysis showed statistically significant correlations between the kyphosis Cobb angle and the amount of EF in all three patient groups.

**Conclusions:**

SEL is a common secondary intraspinal disorder in different types of kyphotic deformities, and surgeons should pay increased attention to this intraspinal anomaly because excessive EF may compress the spinal cord and cause neurological deficits.

## Background

Spinal deformity, including scoliosis and kyphosis, is commonly classified as congenital deformity, degenerative deformity, neuromuscular deformity, idiopathic scoliosis, and deformity caused by syndromes. The coexistence of spinal deformity and intraspinal anomalies has been reported in many previous studies [1–4]. The most obvious and frequently reported anomalies include syringomyelia, Chiari malformation, diastematomyelia, and tethered cord. Apart from these comparatively severe anomalies, which may cause major neurological deficits, several smaller and easily ignored anomalies exist [5, 6], such as diminished spinal cord, narrowed spinal canal, and spinal epidural lipomatosis (SEL).

SEL is characterized as an overgrowth of adipose tissue in the epidural space. SEL is usually asymptomatic; however, if the adipose tissue compresses the spinal cord as the amount increases, it may cause a progressive neurological deficit [7, 8]. Abul-Kasim et al. [6] reported an intriguing finding that epidural lipomatosis is a common comorbidity of Scheuermann disease, which is also known as Scheuermann kyphosis (SK) or juvenile kyphosis, and mainly affects the thoracic or thoracolumbar spine in juveniles [9]. They hypothesized that SEL might play a role in the pathogenesis or progression of SK, as significant correlations were found between the amount of epidural fat (EF) and the severity of kyphosis.

According to our radiological and surgical observation, however, SEL or increased EF may not only occur in SK but also in other spinal kyphotic deformities, which indicates that SEL may not be a primary or pathogenic anomaly but a secondary change. The objective of this study is to test the hypothesis that SEL or increased EF commonly occurs in patients with different types of kyphotic deformities as a secondary disorder.

## Methods

This study was approved by the institutional ethical review board. We included three common kyphotic deformities in the present study: congenital kyphosis (CK), SK, and tuberculotic kyphosis (TK). All patients with these kyphotic deformities who underwent deformity correction surgery at our center during the period from January 2010 to December 2016 were identified from our database. CK is defined as a kyphosis of the spine caused by abnormal vertebral somatogenesis, including failure of either segmentation or formation of vertebrae, or a combination of both [10]. The diagnosis of SK is based on radiographs, and the diagnostic criteria are as follows: kyphosis greater than 40°, disk space narrowing, vertebral end plate irregularity, and anterior wedging of at least 5° in 3 consecutive vertebral bodies [11]. All patients with TK underwent appropriate drug therapy, and the spinal tuberculosis had been cured or silent when the patients visited our spine surgery center. The inclusion criteria consisted of age ≥ 5 years, Cobb angle of scoliosis ≤ 5°, no prior spinal surgery, availability of preoperative anteroposterior and lateral standing X-rays, and availability of preoperative MR images. Patients with potential risk factors for SEL [12–18], such as obesity (body mass index (BMI) ≥ 30), receiving long-term exogenous steroid therapy, Cushing disease, Cushing syndrome, prolactinoma, and hypothyroidism, were excluded from this study. A total of 69 patients (16 with CK, 40 with SK, and 13 with TK) were finally enrolled. Meanwhile, 69 age- and sex-matched control subjects were also enrolled.

**Fig. 1:**
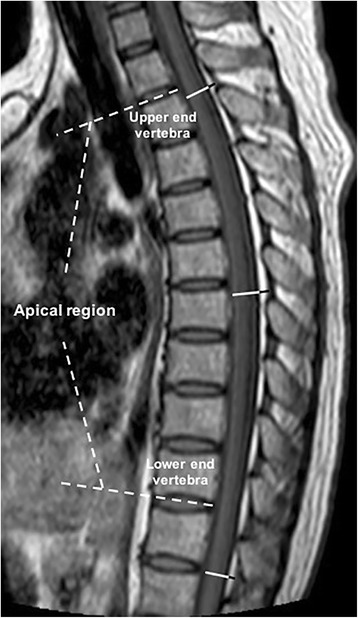
T1-weighted MR image of a normal control. The measurements of the dural sac (DS) are shown in white, and those of the epidural fat (EF) are shown in black. The measurements from proximal to distal level are EF_U_ and DS_U_, EF_A_ and DS_A_, and EF_L_ and DS_L_

**Fig. 2:**
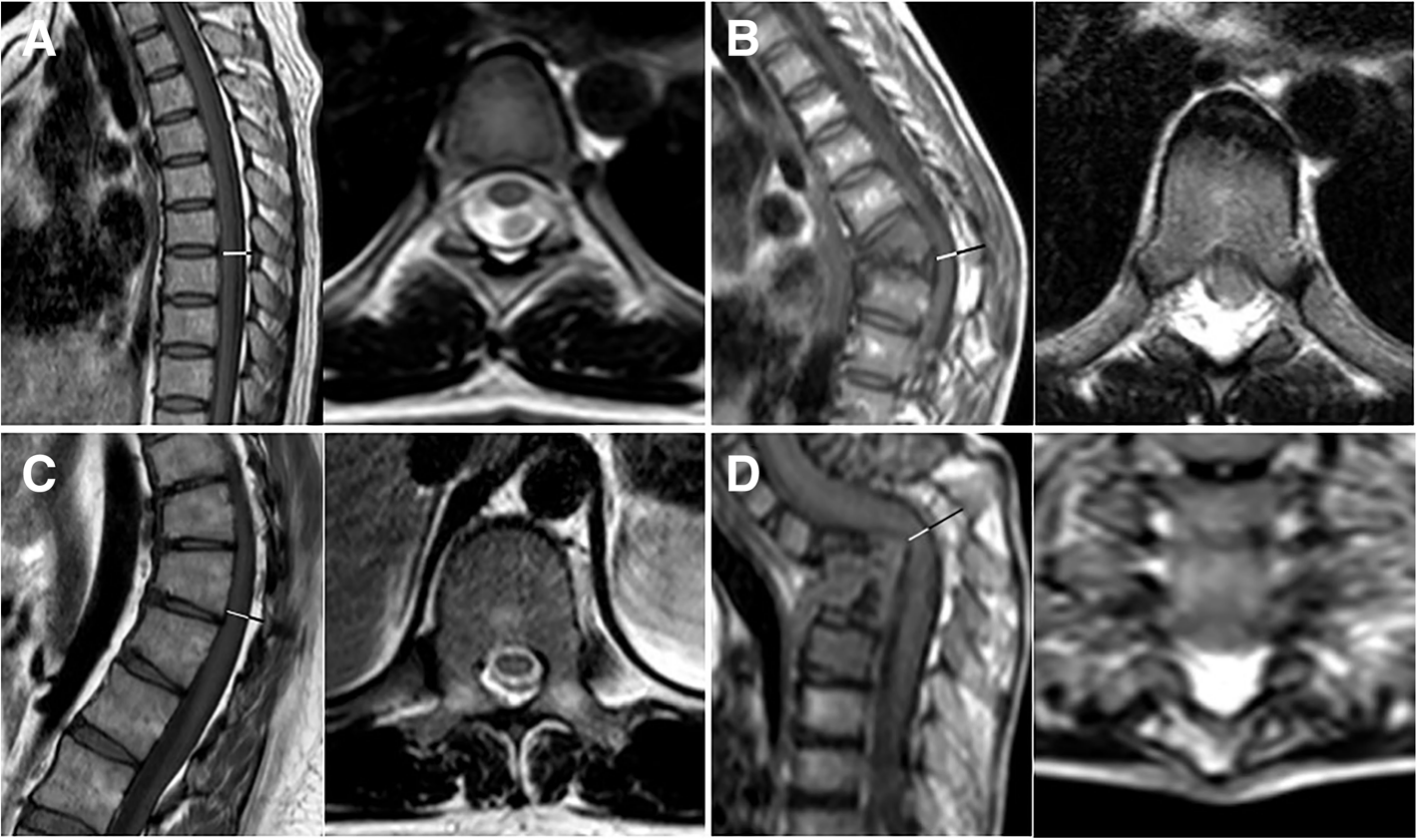
Sagittal and axial MR images of four different individuals included in this study. The measurements of the dural sac (DS) are shown in white, and those of the epidural fat (EF) are shown in black. **a** MRI of a control showing a normal amount of EF at T7 with an EF ratio (EFR_A_) of 0.22. **b** MRI of a patient with congenital kyphosis showing an increased amount of EF at T7 with an EFR_A_ of 0.57. **c** MRI of a patient with Scheuermann kyphosis showing an increased amount of EF at T7 with an EFR_A_ of 0.42. **d** MRI of a patient with tuberculotic kyphosis showing an increased amount of EF at T3 with an EFR_A_ of 0.64

All images were obtained from the Picture Archiving and Communication System and measured with Surgimap (version:2.2, Nemaris Inc.). Spinal kyphosis was assessed by measuring the traditional Cobb angle on standing lateral radiographs as described previously [19]. As T1-weighted MR images provide a clear distinction between EF and the dural sac (DS), EF and DS diameter measurements were performed on sagittal T1-weighted images as described previously [6]. The following measurements were performed in patients with kyphotic deformities: (1) the largest sagittal diameter of EF and the sagittal diameter of the DS in the apical region (EF_A_, DS_A_), one level above the upper end vertebra of the kyphosis (EF_U_, DS_U_), and one level below the lower end vertebra of the kyphosis (EF_L_, DS_L_) (Fig. 1); (2) the average diameter of EF in non-kyphotic regions was calculated as EF_N_ = (EF_U_ + EF_L_) / 2, and the average diameter of the DS in non-kyphotic regions (DS_N_) was calculated in a similar manner; and (3) the sagittal diameter of the spinal canal (SC) was then calculated as the diameter of the EF + diameter of the DS, and the EF ratio (EFR) was calculated as the sagittal diameter of the EF/sagittal diameter of the SC. In the control group, the diameters of the EF and DS were measured at the corresponding levels. All measurements were performed twice with an interval of 4 weeks by the same reader who was blinded to the clinical data. The mean value of the two measurements was used for further analysis. Additionally, the body height and weight of the patients and controls were collected, and the BMI was calculated as weight/height^2^. The neurological status of the patients was also recorded.

The statistical analysis was performed using SPSS 22.0 software (IBM, Armonk, New York). Continuous data are presented as means ± standard deviations (SD). The degree of intra-observer agreement regarding the EF and DS diameter measurements was estimated using the intraclass correlation coefficient (ICC). A paired *t*-test was used to compare the radiographic parameters between the patients and controls. Spearman’s correlation analysis was performed to test the association between two variables. Fisher’s exact test was performed to test the categorical variables. Statistical significance was set at 0.05. Power analysis was performed using PASS (Power Analysis and Sample Size) 11, which showed that minimum 14 patients in CK group, 32 patients in SK, and 8 patients in TK group were needed to detect a significant difference in EF and reach 95% power with alpha at 0.05.

## Results

### Patient demographics

This retrospective study included 69 patients with kyphotic deformities (16 with CK, 40 with SK, and 13 with TK) and 69 control subjects. The average ages of patients with CK, SK, and TK were 13.9 ± 2.7, 18.0 ± 5.5, and 23.8 ± 8.6 years, respectively. The age of the controls was 18.1 ± 6.5 years. The BMI was 18.9 ± 2.9 for CK patients, 24.0 ± 2.7 for SK patients, and 21.7 ± 2.4 for TK patients, and no group was significantly different from the controls (*P* = 0.378, 0.251, and 0.313, respectively). The mean values for kyphosis were 63° ± 20°, 74° ± 9°, and 88° ± 22°, respectively. The apex location varied in different patients. The apex of kyphosis was located in the thoracic spine in 56.3% of CK patients, 85.0% of SK patients, and 69.2% of TK patients (Table 1). Two of the 16 CK patients and 7 of the 13 TK patients had incomplete paraplegia, while the remaining patients had intact neurological status.

**Table 1:** Patient demographics

	Congenital kyphosis	Scheuermann kyphosis	Tuberculotic kyphosis	Control
Number	16	40	13	69
Age	13.9 ± 2.7	18.0 ± 5.5	23.8 ± 8.6	18.1 ± 6.5
Sex (male/female)	6/10	34/6	8/5	48/21
BMI (kg/m^2^)	18.9 ± 2.9	24.0 ± 2.7	21.7 ± 2.4	22.0 ± 3.4
Degree of kyphosis	63° ± 20°	74° ± 9°	88° ± 22°	--
Apex location				
Thoracic spine	9 (56.3%)	34 (85.0%)	9 (69.2%)	--
Lumbar spine	7 (43.7%)	6 (15.0%)	4 (30.8%)	--

### Reliability analysis

ICC analysis showed good reliability in the measurement of the diameter of the EF and DS. The agreement between the two measurements was excellent with an ICC = 0.90 for the sagittal diameter of the EF and an ICC = 0.86 for the sagittal diameter of the DS. The measurement reliability was consistent with a previous study.

### Sagittal diameters of the EF and DS

The mean values and SDs of EF_A_, DS_A_, SC_A_, EFR_A_, EF_N_, DS_N_, SC_N_, and EFR_N_ in patients with kyphotic deformities and controls are shown in Table 2. In the kyphotic region, the EF_A_ was 7.1 ± 1.4 mm for CK patients, 5.4 ± 1.7 mm for SK patients, and 7.6 ± 2.1 mm for TK patients. The differences in EF_A_ between the patients and controls were statistically significant in all three groups (*P* = 0.002, < 0.001, and < 0.001, respectively). Significant differences between the patients and controls were also detected in terms of DS_A_ (*P* = 0.047, 0.009, and 0.002, respectively) and EFR_A_ (*P* = 0.001, < 0.001, and < 0.001, respectively) (Fig. 2). Regarding the diameter of the spinal canal, no differences were found between the patients and controls in any group. In the non-kyphotic region, neither the estimated diameters (EF_N_, DS_N_, and SC_N_) nor the calculated ratios (EFR_N_) showed significant differences between the patients with kyphotic deformities and controls in any group.

**Table 2:** Sagittal diameter of the EF and DS in patients with kyphotic deformities and controls

	CK	CK control	*P*	SK	SK control	*P*	TK	TK control	*P*
EF_A_	7.1 ± 1.4	4.7 ± 1.7	0.002*	5.4 ± 1.7	4.1 ± 1.2	<0.001*	7.6 ± 2.1	3.3 ± 1.3	<0.001*
DS_A_	11.0 ± 2.6	12.4 ± 1.5	0.047*	12.2 ± 2.2	13.4 ± 1.8	0.009*	9.4 ± 2.6	14.0 ± 2.3	0.002*
SC_A_	18.1 ± 2.5	17.1 ± 2.3	0.276	17.6 ± 2.0	17.5 ± 1.8	0.704	17.0 ± 2.3	17.3 ± 2.0	0.790
EFR_A_	0.40 ± 0.10	0.27 ± 0.07	0.001*	0.31 ± 0.10	0.23 ± 0.07	<0.001*	0.45 ± 0.12	0.19 ± 0.07	<0.001*
EF_N_	4.6 ± 1.6	4.6 ± 1.7	0.881	4.2 ± 1.0	3.8 ± 1.1	0.069	4.0 ± 1.6	4.1 ± 1.2	0.862
DS_N_	12.3 ± 1.5	12.2 ± 1.4	0.605	13.5 ± 2.1	13.3 ± 2.1	0.752	13.5 ± 1.8	13.6 ± 2.3	0.918
SC_N_	16.9 ± 2.4	16.9 ± 2.6	0.651	17.6 ± 2.2	17.1 ± 1.8	0.226	17.5 ± 1.9	17.6 ± 1.9	0.854
EFR_N_	0.27 ± 0.07	0.27 ± 0.07	0.809	0.24 ± 0.06	0.22 ± 0.07	0.288	0.23 ± 0.04	0.23 ± 0.08	0.839

*Paired *t*-test

### Prevalence of SEL among patients with kyphotic deformities

According to the diagnostic criteria for SEL previously reported by Borre et al. (EFR > 40%) [20], SEL occurred among 43.8% of CK patients, 20.0% of SK patients, and 84.6% of TK patients and among 6.3%, 2.5%, and 0% of the controls (*P* = 0.019, 0.014, and < 0.001, respectively). It is noteworthy that of all the patients with neurological deficits (2 CK patients and 7 TK patients) were diagnosed with SEL (Table 3).

**Table 3:** The differences between patients with CK, SK, and TK and controls with regard to the previously proposed criteria for SEL

	SEL	Normal	*P*
CK	7 (43.8%)	9 (56.2%)	0.019
Control	1 (6.3%)	15 (93.7%)	
SK	8 (20.0%)	32 (80.0%)	0.014
Control	1 (2.5%)	39 (97.5%)	
TK	11 (84.6%)	2 (15.4%)	<0.001
Control	0 (0%)	13 (100%)	

Data are presented as the number of patients

### Correlation analysis

Spearman’s correlation analysis showed a significant association between the degree of kyphosis and EF_A_ in patients with CK, SK, and TK (*P* = 0.020, 0.014, and 0.011, respectively). The correlation between the degree of kyphosis and EFR_A_ was also statistically significant in SK (*P* = 0.029) and TK patients (*P* = 0.013), and it was marginally significant in CK patients (*P* = 0.052). There were no statistically significant correlations between the age, sex or BMI on one hand and EF_A_ or EFR_A_ on the other hand.

## Discussion

SEL was first reported by Lee et al. [21] in 1975 in a patient who had undergone kidney transplantation. With the development of radiographic technology, especially the wide use of MRI, hundreds of SEL cases have been reported, and it is no longer a rare condition. SEL in patients with spinal deformity, however, has rarely been reported [22–25]. It was first reported by E Kurt and SH Bakker-Niezen [24] in 1995. In that case, SEL was found in a 52-year-old male with neurogenic claudication and lumbar scoliosis. Miyakoshi N et al. [25] reported another case in which a 75-year-old female with lumbar kyphosis, which was caused by osteoporotic vertebral fractures, was found to have lumbar SEL. Abul-Kasim et al. [6] conducted the first and only retrospective study and found that SEL is a common imaging feature in SK. In that study, the authors included 29 SK patients and 58 controls. Among the 29 patients, 12 (41%) fulfilled their proposed diagnostic criteria for SEL (EF > 6 mm and EFR > 0.33), while only 2 (3%) among the controls fulfilled these criteria. A positive correlation between the kyphosis degree and the amount of the EF was also detected. That study included only SK patients; thus, further study was required to clarify the relationship between SEL and spinal kyphosis.

In the present study, we included patients with CK, SK, and TK, which are three common types of spinal kyphosis. Increased epidural adipose tissue was found not only in SK patients, as reported in a previous study [6], but also in CK and TK patients. Furthermore, the occurrence of SEL was also significantly higher in all three patient groups than in controls. Our findings, for the first time, demonstrated that an increased amount of EF or SEL are common intraspinal anomalies in different types of spinal kyphotic deformities. Interestingly, in another study, the EF thickness was shown to decrease in the thoracic spine in patients with adolescent idiopathic scoliosis (AIS) [26]. The finding that thoracic hypokyphosis is an important characteristic of AIS and may play a part in the pathogenesis of scoliosis [27–29] indirectly supports our results. Another intriguing finding of the present study is that the increase of EF occurred in both the kyphotic region (though not statistically significant) and the normal region of the spine in SK patients, while it only occurred in the kyphotic region in CK and TK patients. A possible explanation is that the kyphotic deformity usually involves fewer vertebral levels and is more pronounced in CK and TK patients than in SK patients. In other words, SK is less of a regional spinal deformity than CK and TK.

Furthermore, Spearman’s correlation analysis showed statistically significant correlations between the degree of kyphosis and the EF_A_ and EFR_A_ in all three patient groups, which indicated a potential causal relationship between SEL and spinal hyperkyphosis. Abul-Kasim et al. [6] hypothesized that SEL, as a primary anatomical change, might play a role in the pathogenesis of spinal kyphotic deformity (e.g., SK). Our study, however, demonstrated that SEL should be a secondary rather than a primary change in spinal kyphotic deformities, as it occurred in both congenital and acquired spinal kyphotic deformities. The hyperkyphotic spinal column compresses the DS, which may leave room for the EF. Nevertheless, the molecular mechanism is still unknown, and further studies are required. However, although SEL is a secondary change in spinal kyphotic deformities, it may in turn have an effect on spinal kyphosis. The increased EF might induce a more severe kyphosis to create a larger space for the spinal cord, which is similar to the condition of patients with lumbar spinal stenosis who bend forward to relieve symptoms.

The potential importance of this observation requires attention and cannot be ignored in clinical practice. The causes of neurological deficits in patients with spinal deformity could be multifactorial, and the most widely recognized factors include primary diseases, spinal cord traction induced by deformity, and intraspinal anomalies. Our study added another potential factor, SEL, which was previously ignored. Excessive EF in the spinal canal may compress the spinal cord and cause mild or severe neurological symptoms [8, 30, 31]. In the present study, two CK patients and seven TK patients exhibited incomplete paraplegia, and all of these patients met the diagnostic criteria of SEL. Patients with increased EF, either mild or severe, are at higher risk of compression of the spinal cord or its blood supply. In all three patient groups, the DS_A_ was significantly smaller than that in the controls, which indicated compression exerted by EF on the spinal cord. However, no SK patients showed any neurological deficits, possibly due to the milder degree of SEL than in CK and TK patients. The risk of neurological deficit may arise after kyphosis correction surgery, as the spinal canal will become narrower due to the contraction of spinal ligaments, e.g., the posterior longitudinal ligament and ligamentum flavum. Thus, removal of some of the excessive epidural adipose tissue may be helpful to achieve an improved neurological status.

The present study has several limitations. The first is the retrospective nature of the study. The second is the small numbers of patients (especially CK and TK patients) due to the high prevalence of the coexistence of kyphosis and scoliosis and the strict inclusion criteria (Cobb angle of scoliosis ≤ 5°). Third, we only measured the diameter of EF on the sagittal plane due to the lack of T1-weighted axial MR images, as the T1-weighted axial MR scan was not a routine procedure in our center. Fourth, all of the patients included in this study had severe kyphotic deformities and required surgical treatment. Patients with mild deformities were not included. Fifth, the control groups included were not normal populations. However, none of the controls had spinal deformities, and the diseases of the controls should not influence the measurement of EF or the DS.

## Conclusion

In conclusion, this study showed that SEL was a common secondary disorder in different types of kyphotic deformities, including CK, SK, and TK. We believe that this finding has important clinical significance. Surgeons should pay increased attention to this secondary disorder, as excessive EF may compress the spinal cord and its venous outflow. This is especially the case after spinal kyphosis correction surgery, as the spinal canal will become narrower due to the contraction of spinal ligaments. We suggest that preoperative spine MRI findings should be carefully evaluated, and removal of excessive EF should be taken into consideration.

## Abbreviations

CK: Congenital kyphosis
DS: Dural sac
EF: Epidural fat
SK: Scheuermann kyphosis
TK: Tuberculotic kyphosis
